# Prognostic implications of autophagy and pyroptosis related genes in oral squamous cell carcinoma: research on bioinformatics and experiments

**DOI:** 10.3389/fonc.2025.1645670

**Published:** 2025-08-29

**Authors:** Leshui Qiao, Binghui Si, Jiangtian Xiao, Xiaoyu Song

**Affiliations:** ^1^ School of Stomatology, Jilin University, Changchun, China; ^2^ School of Basic Medical Sciences, Harbin Medical University, Harbin, China; ^3^ Department of Microbiology and Molecular Genetics, Michigan State University, East Lansing, MI, United States; ^4^ Laboratory of New Technologies for Physical Diagnosis and Treatment, School of Life Sciences and Technology, Harbin Institute of Technology, Harbin, China

**Keywords:** autophagy, pyroptosis, oral squamous cell carcinoma, prognosis, BAK1, biomarker

## Abstract

**Objective:**

Oral squamous cell carcinoma (OSCC) is associated with poor prognosis, underscoring the urgent need to identify biomarkers that can predict patient outcomes and guide personalized treatment strategies. This study analyzes the prognostic potential of autophagy and pyroptosis-related genes in OSCC by bioinformatics approaches.

**Methods:**

Clinical data and gene expression profiles for OSCC were obtained from the TCGA database, while autophagy and pyroptosis-related genes were retrieved from the GeneCards database. A prognostic model based on these genes was constructed using the LASSO Cox regression method. The performance of the model was evaluated through ROC curve analysis and survival analysis. Gene set enrichment analysis (GSEA) was performed to explore the functional pathways associated with these genes, and immune infiltration was assessed through immune profiling. The independent prognostic value of the genes included in the model was evaluated using multivariate Cox regression and nomogram analyses.

**Results:**

Eight autophagy and pyroptosis-related genes (BAK1, ATG5, VEGFA, GABARAPL1, RSL1D1, CYCS, TOMM20, and PGAM5) were found to have significantly lower overall survival (OS) in the high-expression group compared to the low-expression group in OSCC tissues. GSEA revealed that these genes were involved in pathways such as the Initial Triggering of Complement, the Role of Phospholipids in Phagocytosis, CD22-Mediated BCR Regulation, and the Role of Lat2/NATL/LAB in Calcium Mobilization. Furthermore, BAK1 and GABARAPL1 were positively correlated with immune cell infiltration in OSCC, while the other six genes showed negative correlations with immune infiltration. Notably, BAK1 and GABARAPL1 were identified as independent prognostic factors for OSCC, with BAK1 showing particularly strong diagnostic predictive potential. Functional assays revealed that silencing BAK1 in OSCC cell lines (SCC-15 and CAL-27) significantly inhibited cell proliferation and migration *in vitro*.

**Conclusions:**

Our findings suggest that the high expression of eight autophagy and pyroptosis-related genes in OSCC correlates with poor prognosis. In particular, BAK1 emerges as an independent prognostic marker with high diagnostic potential. These results offer promising insights into the development of gene-targeted therapies for OSCC, with BAK1 representing a potential therapeutic target.

## 1 Introduction

Oral squamous cell carcinoma (OSCC) originates in the oral mucosa, comprising about 90% of oral malignant tumors and ranking among the most prevalent head and neck malignancies (1, 2).According to the Global Cancer Observatory (GCO), 389,846 cases of OSCC were reported globally in 2022, with Asia experiencing a particularly high incidence (3). Despite advancements in treatments, including surgery, chemotherapy, radiotherapy, immunotherapy, and targeted therapy, which have somewhat enhanced patient survival rates (4, 5),the prognosis remains unfavorable. Specifically, the five-year survival rate for OSCC stands at only 41%, with a minimal annual increase of less than 1% observed from 2005 to 2017 (6–8). Early diagnosis and detection of OSCC can improve survival rates to 80-90% (9, 10). Therefore, it is crucial not only to focus on early diagnosis but also to identify biomarkers that can predict patient prognosis and guide personalized treatment strategies.

Current studies indicates that the regulatory relationship between autophagy and pyroptosis occupies a fundamental position in Tumor Progression and Drug Resistance. Autophagy is a basic biological process in cells. It is a process of degrading biological macromolecules and damaged organelles through lysosomes, including macroautophagy, microautophagy and molecular chaperone-mediated autophagy (CAM) (11). Alleviating cell stress and nutrient starvation (12), is essential to maintain the stability of the intracellular environment. Pyroptosis, a programmed cell death mechanism, is mediated by the gasdermin (GSDM). This process itself triggers an inflammatory response, releases damage-associated molecular patterns (DAMPs) and inflammatory cytokines such as IL-1β and IL-18, and causes immune cell infiltration (13). Pyroptosis interacts with immune cells, which can influence the tumor microenvironment. Even if only a small fraction of the tumor cells are focalized, it is sufficient to clear the entire tumor (14–17). Autophagy can negatively regulate pyroptosis by regulating related signaling pathways, and can also mediate the autophagic degradation of pyroptosis-related proteins by acting on related proteins such as TRIM20, IRGM, USP5, LGALS9, USP18, etc. (18–23). Interestingly, activation of pyroptosis pathways can promote autophagy. For example, (pro-) caspase-1 protein, NLR components and potassium efflux can induce autophagy (24). In addition, the regulation of autophagy on pyroptosis involves the innate immune system. In the innate immune system, mitochondrial damage can activate the cGAS-STING signaling pathway, thereby inducing pyroptosis and autophagy. This may be a key mechanism mediating acute to chronic inflammation and inflammation-cancer transformation in non-tumor tissues (25–29). However, the increase of autophagy level in tumor cells is often accompanied by the decrease of sensitivity to chemotherapy, radiotherapy and immunotherapy, which leads to autophagy-related drug resistance. Autophagy has also become a crucial target for drug resistance intervention, which is worth studying (30–32). Growing evidence suggests that inhibiting autophagy can improve treatment outcomes in patients with advanced cancers. Based on the negative regulation of autophagy on pyroptosis, the use of doxorubicin or paclitaxel to inhibit autophagy to promote pyroptosis has emerged as novel approaches to overcome drug resistance (33, 34).

In recent years, prognostic models based on autophagy-related genes have demonstrated precise predictive capabilities in various common cancers (35–38). Correspondingly, pyroptosis-related genes have also successfully established well-performing prognostic models in cancer species (39–42). Furthermore, numerous studies have shown that modulation of autophagy might serve as a new therapeutic target for OSCC, with evidence indicating that autophagy is related to various biological processes in OSCC, such as proliferation, angiogenesis, and drug resistance (43–46). Compared with adjacent tissues, OSCC has a high degree of autophagy activity. The double expression of ATG5 and BECN1 is a poor prognostic indicator of OSCC (47). However, autophagy seems to be a ‘ double-edged sword ‘ in OSCC. Some researchers believe that autophagy can promote apoptosis and thus play a role in inhibiting tumors, while others argue that it increases the stemness and malignancy of OSCC cells (48, 49). Recent bioinformatics studies have also elucidated the prognostic role of pyroptosis-related genes in OSCC immunotherapy (50, 51).

Overall, both autophagy and pyroptosis are closely linked to the inflammatory response, immune response, drug resistance, and prognosis in OSCC. However, it is worth noting that autophagy and pyroptosis are not two independent processes. On the contrary, there is a very complex regulatory relationship between them. As mentioned above, most current research tends to investigate autophagy or pyroptosis separately, focusing on their individual roles in tumor development and prognosis. Yet, the intersecting genes between autophagy and pyroptosis may furnish fresh perspectives on the fundamental biological mechanisms of OSCC and identify novel therapeutic targets. This intersection also suggests that the complex interplay and regulation between autophagy and pyroptosis may have an overarching effect on the occurrence and development of OSCC. Furthermore, examining these interactions in detail may reveal the physiological and pathological effects of these processes in OSCC. Research that treats autophagy and pyroptosis as distinct and independent processes could potentially overlook critical information related to treatment and prognosis. One study has combined autophagy and pyroptosis intersection genes to construct a prognostic model for renal cell carcinoma (52). However, it is not clear whether autophagy and pyroptosis related genes can play a role in predicting the prognosis of patients and guiding individualized treatment in OSCC. Therefore, we utilized bioinformatics to establish a OSCC prognosis model based on autophagy and pyroptosis related genes. We analyzed gene expression, immune correlation and gene set enrichment, so as to identify independent prognostic markers of OSCC and evaluate their accuracy. Additionally, we performed gene knockdown experiments in two OSCC cell lines for further *in vitro* verification. Our investigation, which includes immune infiltration analysis and pathway exploration, aims to elucidate the potential mechanisms through which autophagy and pyroptosis related genes predict prognosis in OSCC patients. It is hoped that these genes may contribute to more accurate patient-specific prognostic assessments and guide individualized treatment.

## 2 Materials and methods

### 2.1 Data collection

The expression profile data of prognostic-related genes were derived from The Cancer Genome Atlas (TCGA). The RNA sequencing data originating from the TCGA-HNSC (The Cancer Genome Atlas - Head and Neck Squamous Cell Carcinoma) project underwent processing via the STAR alignment pipeline. Subsequently, data in the TPM (Transcripts Per Million) format, in conjunction with pertinent clinical information, were extracted for further examination. Data related to prognosis were sourced from the article of Liu et al., 2018 (53). Data normalization was performed using log2(x+1) transformation. All analyses and visualizations were conducted in version R 4.2.1. Batch fitting of Cox proportional hazards models using the “survival” package (version 3.3.1) was performed to identify OSCC prognosis-related genes with statistical significance. Autophagy and pyroptosis related genes were sourced from the GeneCards database (accessed on 25 February 2025).In this process, the pathways of ‘autophagy’ and ‘pyroptosis’ were searched separately within the database, with the former yielding 9,933 genes and the latter 877 genes. Genes that meet the criteria of hazard ratio (HR) > 1, 95%, and confidence interval (CI) > 0.4, and p < 0.05 were considered significantly related with autophagy and pyroptosis.

### 2.2 Identification of autophagy and pyroptosis related genes and prognostic model construction

First, the identification of OSCC prognostic genes was performed using the “survival” package (version 3.3.1) to conduct batch survival regression analyses, applying Cox regression with overall survival (OS) as the endpoint. Genes demonstrating statistical significance (p < 0.05) were selected, yielding 2,957 prognosis-associated candidates. Subsequently, an intersection was taken between the autophagy, pyroptosis, and OSCC prognostic-related gene datasets extracted during the data collection process. The results were visualized by ‘ggplot2 ‘ (version 3.4.4) and ‘ennDiagram ‘V (version 1.7.3) to obtain 16 co-expressed genes. LASSO regression analysis was applied to these genes to identify candidates with robust prognostic significance, thereby constructing a prognostic model.

### 2.3 Evaluation of prognostic model performance

Based on the median risk score, patients in the TCGA dataset were stratified into high- and low-risk groups. The discriminatory capacity of the model was visualized using risk score plots, heatmaps, and scatter plots, generated with the “ggplot2” R package (version 3.4.4). Survival analysis was performed by fitting a Cox proportional hazards regression model following the proportional hazards assumption test, using the “survival” package. The results were visualized with the “survminer” (version 0.4.9) and “survival” (version 3.2.10) R packages, and were presented as Kaplan–Meier (KM) survival curves. Time-dependent ROC curve analysis was conducted using the “timeROC” R package (version 0.4), and the results were visualized with “ggplot2” (version 3.4.4).

### 2.4 Validation of prognostic features of the model

To evaluate the prognostic performance of individual genes included in the model, diagnostic ROC analysis was conducted using the “pROC” R package (version 1.18.0), and the results were visualized using “ggplot2” (version 3.4.4). To determine whether each gene in the model serves as an independent prognostic factor and to visualize clinical features associated with prognosis, multivariate Cox regression analysis was performed. Corresponding survival outcomes were represented by Kaplan–Meier survival curves. Subsequently, a prognostic nomogram and calibration curves were constructed using the “rms” (version 6.3-0) and “survival” (version 3.2.10) packages. The nomogram integrated the multivariate analysis results to quantitatively predict patient survival probabilities, while calibration curves were used to assess the concordance between predicted and observed outcomes. Finally, a composite risk score was calculated by integrating the expression levels of all genes in the prognostic model, using weights derived from LASSO regression. This score was subsequently evaluated via Cox proportional hazards analysis to reduce model dimensionality and confirm its prognostic relevance.

### 2.5 Independent external validation of model predictive robustness

To evaluate the clinical applicability of the TCGA-derived training model, an independent external dataset (GSE41613) from the GEO database (https://www.ncbi.nlm.nih.gov/geo/) was used for external validation. Kaplan–Meier survival analysis and Cox regression analysis were conducted in the validation cohort, and time-dependent ROC curves were generated to further assess the predictive performance of the model.

### 2.6 Expression analysis of autophagy and pyroptosis related genes

The expression differences of 8 autophagy and pyroptosis related genes screened by LASSO regression were studied, presented in violin plots and paired sample plots. The samples were stratified into high- and low-expression subgroups based on the median expression threshold of the target gene, enabling subsequent analytical investigations. Data visualization was conducted using the “ggplot2” package (version 3.4.4), and statistical analysis was conducted using the “stats” package (version 4.2.1) and the “car” package (version 3.1-0).To visualize expression profiles of the eight candidate genes across OSCC cell lines, we generated an interactive heatmap leveraging transcriptomic data from the DepMap database (accessed on 21 July 2025), implemented through the ShinyThor computational platform (54). The intracellular localization of each gene was from the Genecards website to further clarify its subcellular distribution.

### 2.7 Gene set enrichment analysis

To elucidate the underlying molecular mechanisms by which eight autophagy- and pyroptosis related genes contribute to the tumorigenesis and progression of OSCC, we conducted GSEA to infer their downstream KEGG signaling pathways. GSEA, a computational approach, systematically evaluates whether predefined gene sets display statistically significant and concordant differences in expression patterns between two distinct biological states. Unlike GO and KEGG, which focus on differentially expressed genes with explicit threshold criteria, GSEA does not require predefined differential gene thresholds. This approach reduces the likelihood of overlooking genes with insignificant differential expression but critical biological significance, as well as valuable information regarding gene regulatory networks and functional annotations. GSEA was performed using the MSigDBCollections gene set database for Homo sapiens, with the reference gene set c2.cp.all.v2022.1.Hs.symbols.gmt (3050). Molecular IDs were converted prior to analyses, and GSEA was executed using the “clusterProfile” package (version 4.4.4). Enrichment results were filtered by FDR (q-value) < 0.25 and p.adjust < 0.05, and the top 10 gene sets with the highest absolute enrichment scores were identified. The visualization process was completed using the “ggplot2” package (version 3.4.4).

### 2.8 Immune infiltration analysis

Given the close association between autophagy, pyroptosis and immune cell infiltration and inflammation, the relationship between 8 autophagy and pyroptosis related genes and 24 immune cell types was investigated. The immune cell subsets characterized in this analysis comprised activated dendritic cells (aDC), B lymphocytes, CD8^+^ T cells, cytotoxic T lymphocytes (CTLs), conventional dendritic cells (cDC), eosinophils, immature dendritic cells (iDC), macrophages, mast cells, neutrophils, NK CD56bright cells, NK CD56dim cells, natural killer (NK) cells, plasmacytoid dendritic cells (pDC), T lymphocytes, T helper (Th) cells, central memory T (Tcm) cells, effector memory T (Tem) cells, follicular helper T (Tfh) cells, gamma delta T (Tgd) cells, Th1-polarized cells, Th17-polarized cells, Th2-polarized cells, and regulatory T (Treg) cells. The single-sample gene set enrichment analysis (ssGSEA) algorithm from the “GSVA” package (version 1.46.0) was applied, utilizing marker genes for the 24 immune cell types provided (55, 56). Specifically, Spearman correlation analysis was performed between the primary variables and the immune infiltration matrix. Visualization of the correlation results was achieved via lollipop plots generated by the “ggplot2” package (version 3.4.4).

### 2.9 Cell culture and transfection

The human OSCC cell lines SCC-15 and CAL-27 were obtained from Hunan Fenghui Biotechnology Co., Ltd. Both cell lines were cultured in DMEM (Gibco, Thermo Fisher, 11965092) supplemented with 10% FBS (Gibco, Thermo Fisher, A5256701), 100 U/ml penicillin, and 100 μg/ml streptomycin (Beyotime, C0222) in a humidified atmosphere at 37°Cwith 5% CO_2_. Transfection experiments were executed with the Superkine™ Lipo3.0 transfection reagent (Abbkine, China) in strict accordance with the manufacturer’s protocol. For functional validation studies, BAK1 was selected as the representative candidate gene. Cells were transfected with BAK1 siRNA when reaching optimal density under healthy culture conditions. The siRNA sequences were designed and chemically synthesized by Genepharma Co., Ltd. (Shanghai, China), with their specific sequences detailed in the following section.

siRNA-1:sense:5’-GCCUAUGAGUACUUCACCATT-3’;antisense: 5’-UGGUGAAGUACUCAUAGGCTT-3’.siRNA-2:sense:5’-GCUUCGUGGUCGACUUCAUTT-3’;antisense:5’-AUGAAGUCGACCACGAAGCTT-3’.siRNA-3:sense:5’-GUCCCAUCCUGAACGUGCUTT-3’;antisense:5’-AGCACGUUCAGGAUGGGACTT-3’.

### 2.10 Western blot analysis

Cells were harvested 48 hours after siRNA transfection for protein extraction. Lysis was performed using RIPA buffer (Thermo Fisher Scientific) supplemented with protease inhibitors (Roche cOmplete), followed by centrifugation at 12,000 × g for 15 minutes at 4 °C to remove debris. Protein concentrations were quantified using the BCA Protein Assay Kit (Pierce), and equal amounts of protein (25 µg per sample) were separated by SDS–PAGE and transferred to PVDF membranes (Millipore).Membranes were blocked in 5% non-fat dry milk prepared in TBS-T for 1 hour at room temperature and then incubated overnight at 4°C with primary antibodies: rabbit anti-BAK1 (Thermo Fisher Scientific, A305-202A, 1:500) and mouse anti-GAPDH (Cell Signaling Technology, #5174, 1:5000), which served as a loading control. After three washes with TBS-T, membranes were incubated with HRP-conjugated secondary antibodies (Jackson ImmunoResearch, 1:5000) for 1 hour at room temperature. Protein bands were visualized using enhanced chemiluminescence (GE Healthcare) and imaged with the ChemiDoc MP Imaging System (Bio-Rad). Densitometric analysis was performed using ImageJ, with BAK1 expression normalized to GAPDH.

### 2.11 Cell growth and proliferation assay

The CCK-8 assay is a high-sensitivity method used to detect cell proliferation and cytotoxicity by measuring cell metabolic activity, which indirectly reflects cell number and viability. Cell proliferation rates were determined using the commercially available Cell Counting Kit-8 (CCK-8) assay (Abmole, Houston, TX, USA). Transfected cells were plated at a density of 2000 cells per well in 96-well culture plates. Following cell adhesion, 10 μL of CCK-8 reagent was immediately added to each well, and the plates were incubated at 37°C for 2 hours. Subsequent CCK-8 assays were conducted at 24, 48, and 72 hours post-plating, with 10 μL of CCK-8 reagent added to each well followed by a 2-hour incubation at 37°C. The absorbance values at 450 nm (OD_450_) were measured using a microplate spectrophotometer (Infinite M200 Pro, Tecan Group, Männedorf, Switzerland). The relative cell growth rate was calculated as follows: Relative cell growth rate = (OD Day1/Day2/Day3)/OD Day0 × 100%.

### 2.12 Cell migration assay

Transwell assays and scratch wound healing assays were utilized to assess cell migratory capacity. Given the migratory characteristics of monolayer adherent cells, the scratch wound healing assay was specifically applied to evaluate this capability. Logarithmically growing cells were seeded into 6-well plates, and upon reaching 90–95% confluence, a linear scratch was generated using a sterile 10 μL pipette tip. The scratched monolayers were then washed with PBS to remove cellular debris, followed by addition of serum-free medium to minimize growth factor interference while preserving cell viability. Plates were incubated at 37°C, and wound closure dynamics were tracked at 0 and 24 hours post-scratch. For quantitative analysis, three representative fields per scratch area were photographed using an inverted microscope. Transwell migration assays were conducted using Corning Transwell chambers (8.0 μm pore diameter, 24-well plate configuration). For the assay, 200 μL of cell suspension was loaded into the upper chamber, while the lower chamber was filled with 600 μL of complete medium containing 20% FBS as a chemoattractant. The transwell system was then placed into a 24-well plate and incubated at 37°C for 24 hours to allow cell migration. Following incubation, non-migrated cells on the upper membrane surface were carefully washed with PBS and removed using sterile cotton swabs. The migrated cells on the lower membrane surface were fixed with 4% paraformaldehyde for 20 minutes, stained with 0.1% crystal violet solution, and air-dried at room temperature. Finally, the stained membranes were examined under a light microscope, and five randomly selected fields per membrane were photographed to quantify the number of transmigrated cells.

### 2.13 Statistical analysis

Biological experimental data underwent statistical analysis via GraphPad Prism software (version 10.2; GraphPad Software Inc., San Diego, CA, USA). Continuous variables were quantified and expressed as mean ± SD. For pairwise comparisons between independent groups, the Student’s t-test was applied; whereas for paired sample comparisons, the paired t-test was utilized. To analyze differences among multiple groups, one-way or two-way analysis of variance (ANOVA) was conducted. Statistical significance was defined as a p-value < 0.05.

## 3 Result

### 3.1 Identification of autophagy and pyroptosis related genes

**Figure 1:**
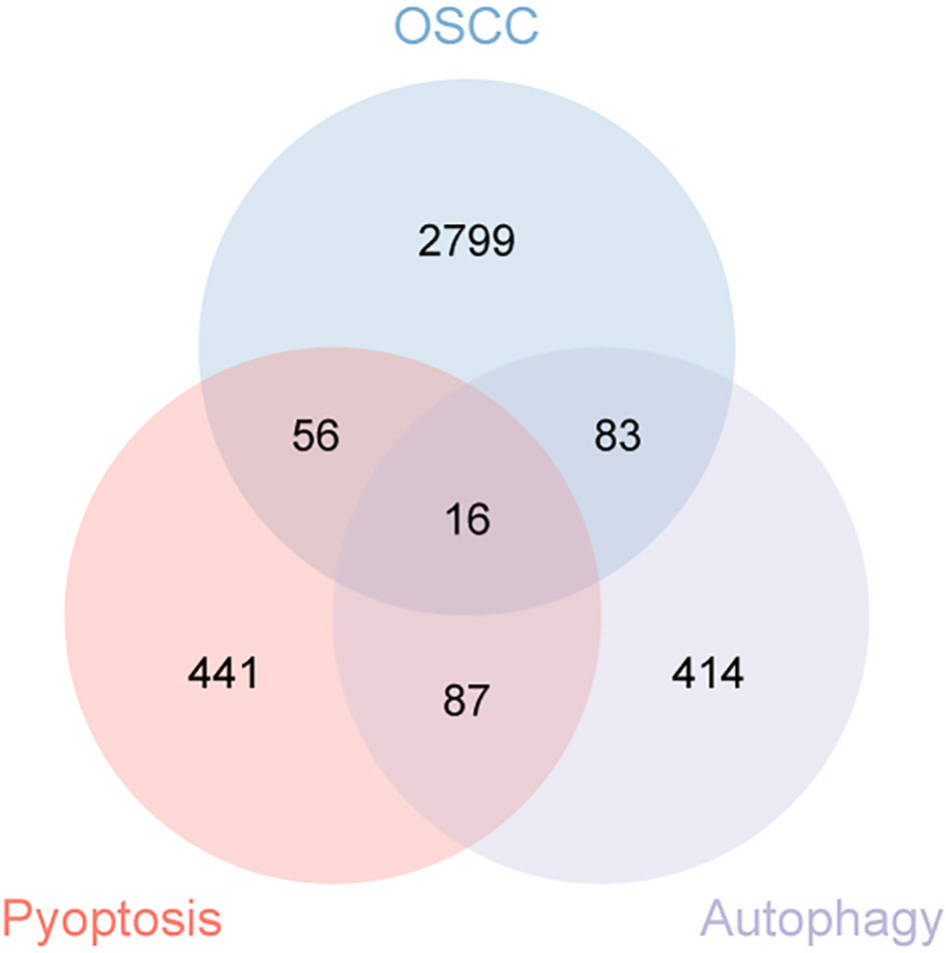
Venn diagram illustrating the co-expressed genes from autophagy, pyroptosis, and OSCC.

**Figure 2:**
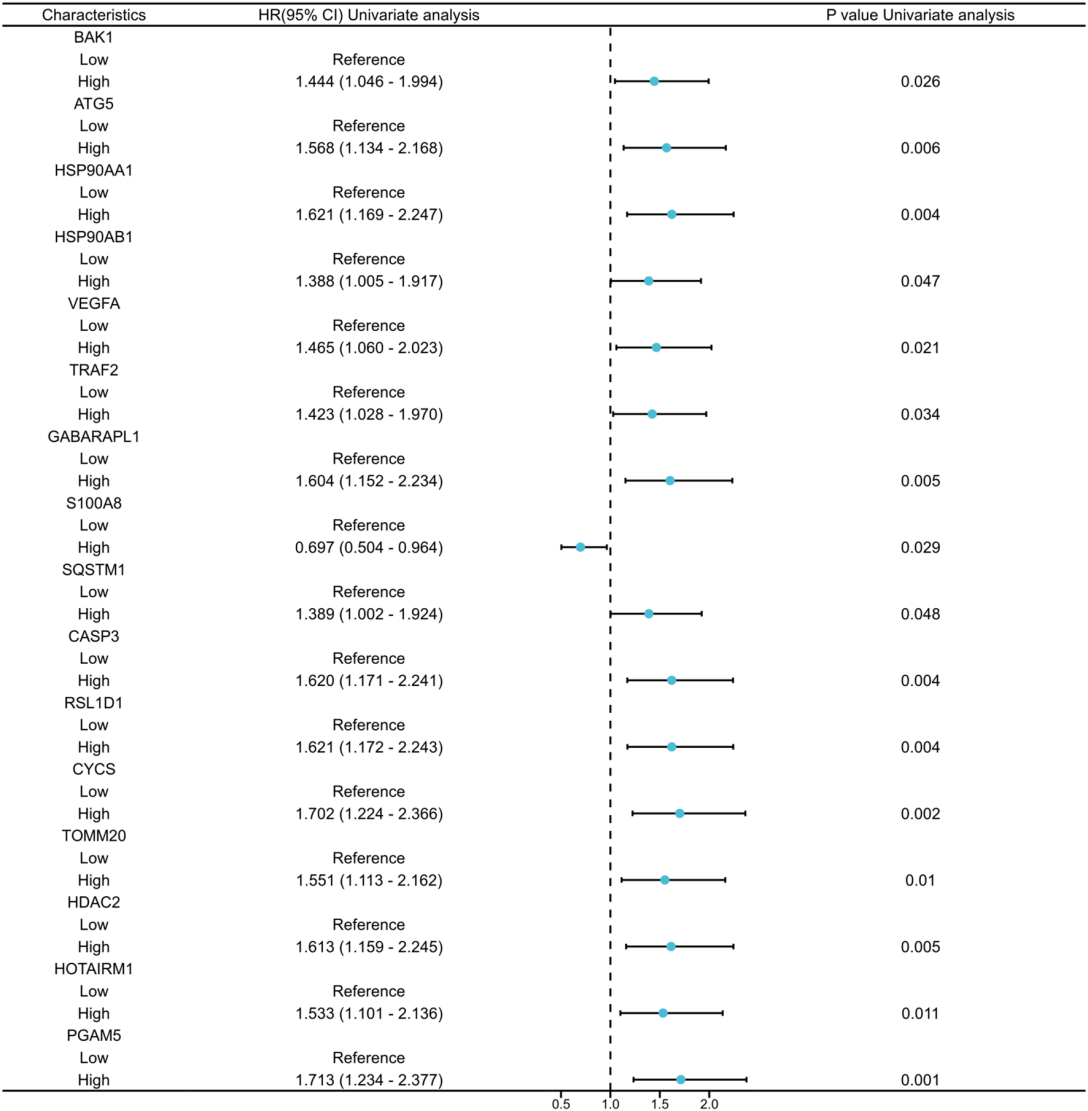
Univariate Cox regression analysis of the 16 co-expressed genes.

A total of 336 OSCC samples were identified from the TCGA database, along with RNA-seq data and corresponding clinical data from 32 normal controls sourced from GTEx. A survival regression analysis was performed on these datasets, with a p-value threshold of <0.05, yielding 2,957 OSCC prognostic-related genes. A search for “autophagy” and “pyroptosis” was conducted using the GeneCards database, and the top 600 genes were selected based on their relevance score, ranked from highest to lowest. The intersection of the three datasets resulted in 16 common genes (**Figures 1**, **2**).

### 3.2 Prognostic model development

**Figure 3:**
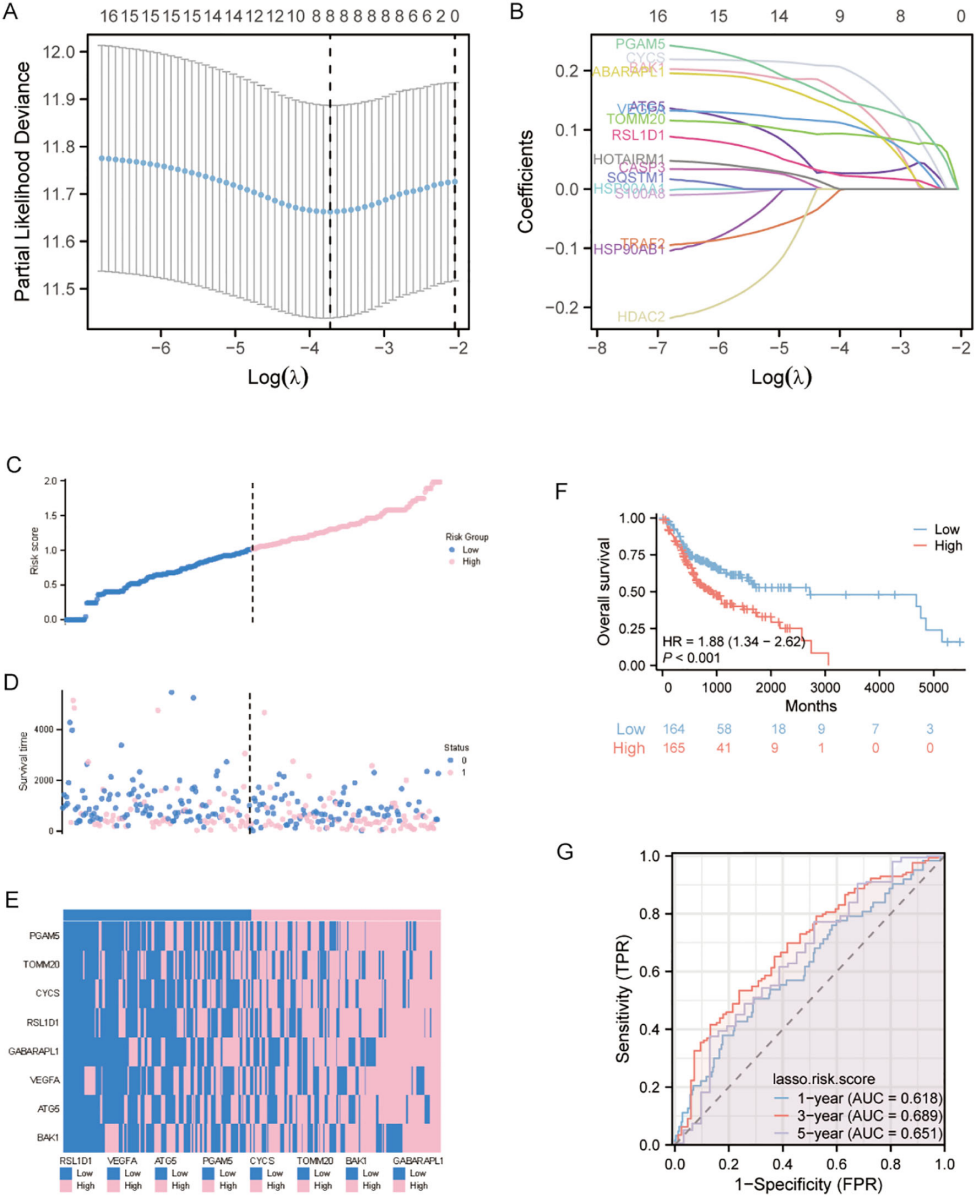
LASSO-Based prognostic model development and validation **(A)** Ten-fold cross-validation curve demonstrating optimal parameter selection (λ) for the LASSO regression model. **(B)** Trajectory plot of variable coefficients during LASSO regularization, showing feature selection dynamics as the penalty parameter increases. **(C)** Risk stratification profile displaying the distribution of calculated risk scores among OSCC patients, with vertical dashed line indicating the optimal cutoff separating high- and low-risk cohorts. **(D)** Survival status visualization plotting individual patient outcomes against corresponding risk scores, with color-coding reflecting vital status (alive: blue; deceased: pink).**(E)** Unsupervised clustering analysis of signature gene expression patterns across patient samples, using a bicolor gradient (pink: z-score >0; blue: z-score <0) to represent normalized expression levels.**(F)** Kaplan-Meier estimator curves comparing overall survival probability between risk groups. **(G)** Temporal ROC analysis evaluating model performance at clinically relevant endpoints (1-, 3-, and 5-year survival).

The 16 autophagy and pyroptosis related genes identified were subjected to LASSO regression model to optimize model parsimony and mitigate collinearity and overfitting (**Figure 3A**). Methodologically, we implemented 10-fold cross-validation and selected λ_min_ as the optimal regularization threshold. This analysis resulted in a final prognostic model containing 8 genes: BAK1, ATG5, VEGFA, GABARAPL1, RSL1D1, CYCS, TOMM20, and PGAM5 (**Figure 3B**).

### 3.3 Prognostic model evaluation

To characterize the distribution of risk scores and verify the robustness of the predictive model, we systematically stratified the samples into high- and low-risk subgroups using the median risk score as the critical cutoff value. Three visualization methods were employed: risk score curves for gene contributions, scatter plots of survival time distributions, and heatmap depicting differential expression of prognostic genes between risk groups. Analyses revealed that the high-risk cohort displayed a statistically significant elevation in risk scores and exhibited inferior overall survival compared with the low-risk group (**Figures 3C, D**). Additionally, heatmap visualization demonstrated a robust positive association between high model expression levels and high-risk stratification (**Figure 3E**). Kaplan-Meier survival curves comparing high- and low-risk groups demonstrated significantly reduced overall survival (OS) in the high-risk cohort (**Figure 3F**), corroborating this association.

To assess model accuracy, prognostic ROC curves, a nomogram, and calibration curves were constructed. The area under the ROC curve (AUC) for 1-, 3-, and 5-year survival predictions were 0.618, 0.689, and 0.651 (**Figure 3G**).

### 3.4 Independent prognostic value assessment of autophagy and pyroptosis related genes and other potential risk factors

**Figure 4:**
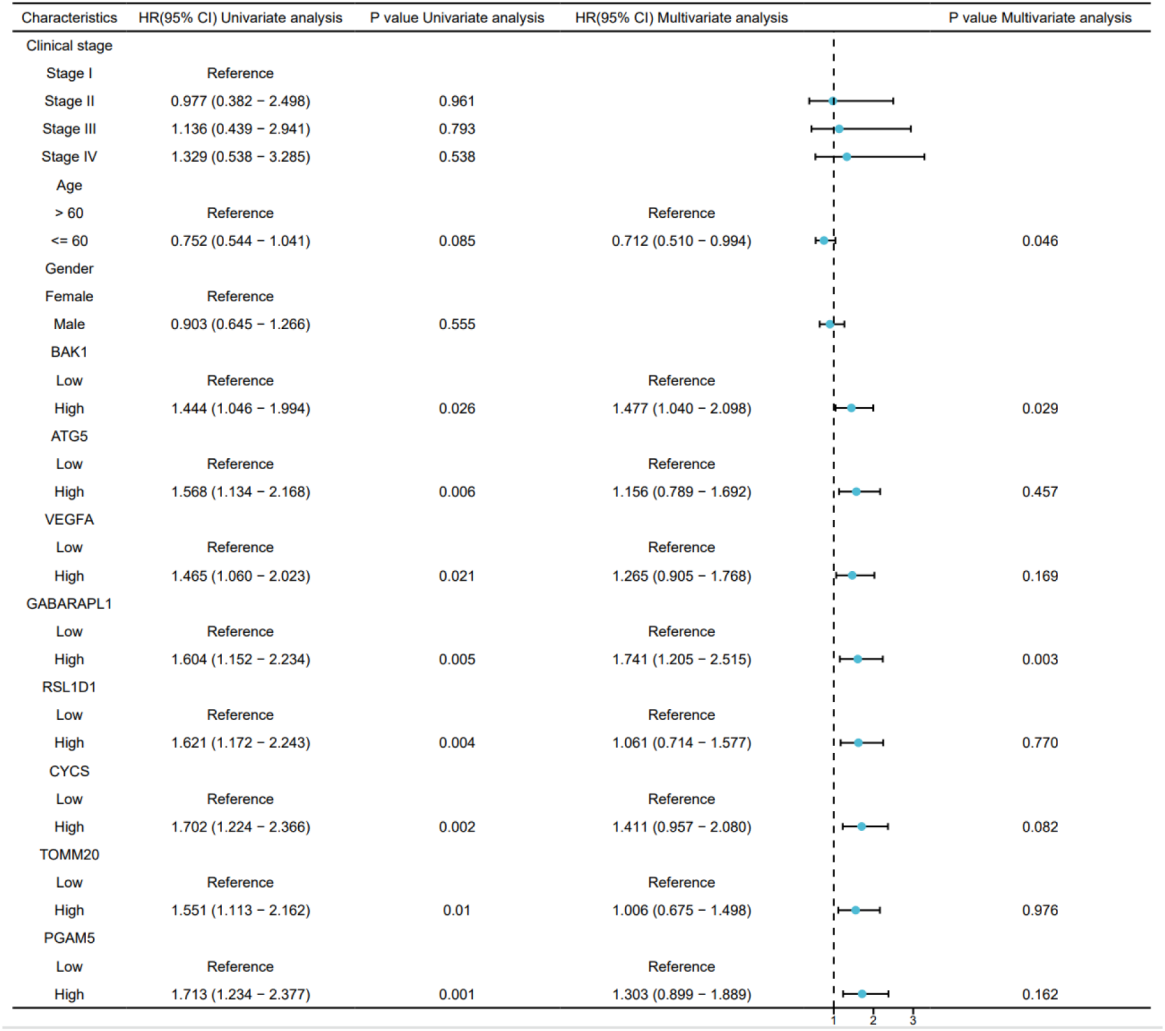
Univariate and multivariate Cox regression analyses of the combined risk score and clinical variables.

**Figure 5:**
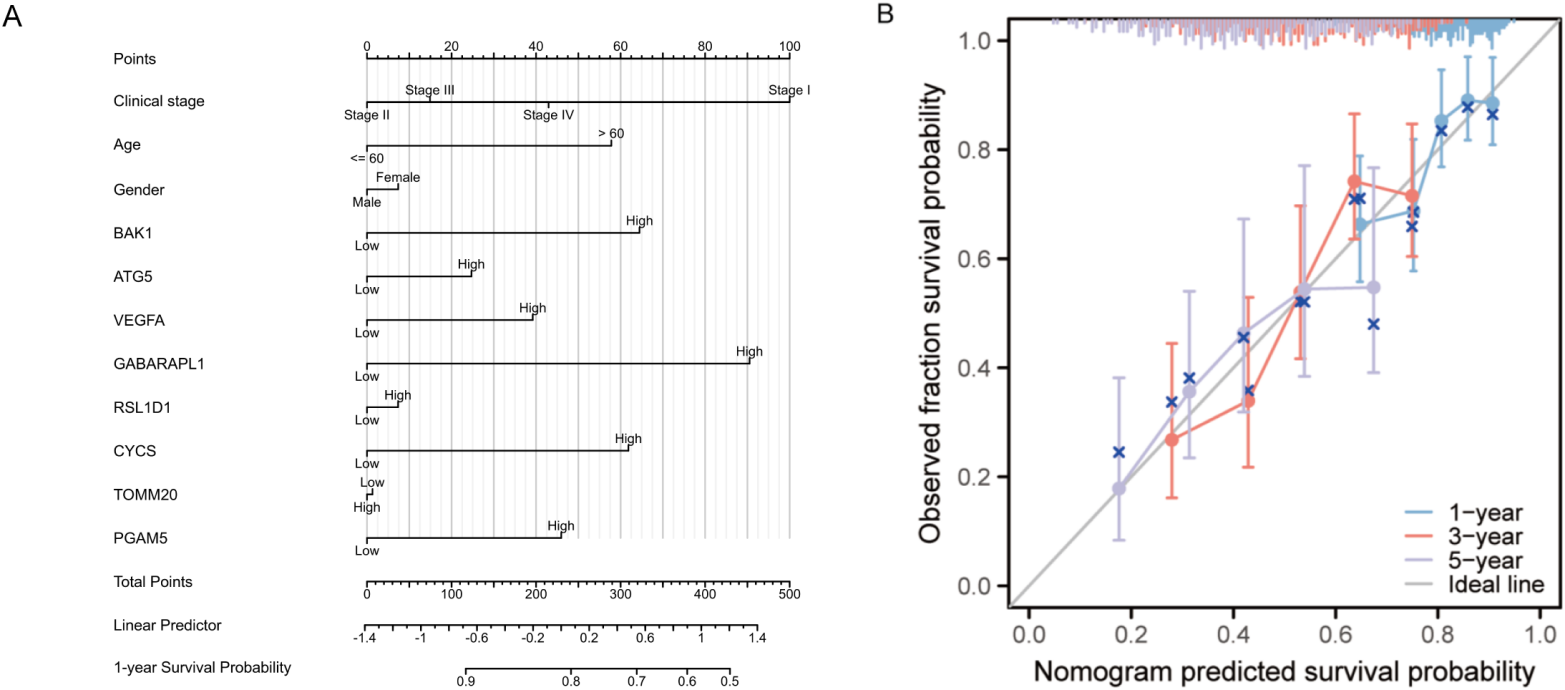
**(A)** Prognostic nomogram integrating key clinical characteristics and prognostic model genes. **(B)** Calibration curve assessing the nomogram’s accuracy in predicting survival probabilities at 1, 3, and 5 years.

**Figure 6:**
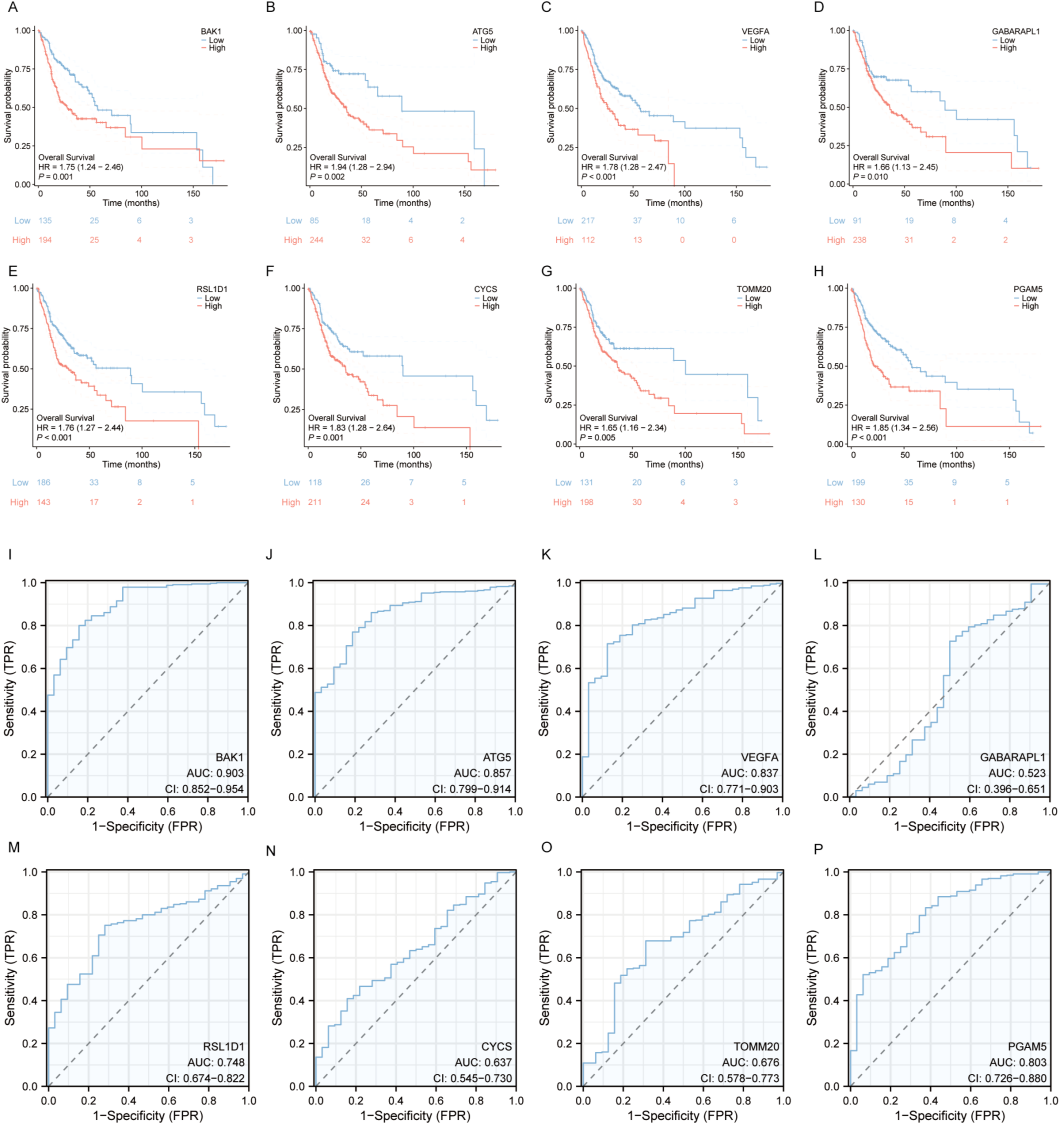
Survival Analysis and Diagnostic ROC Curves of the 8 Prognostic Model Genes in OSCC. **(A–H)** Relationship between the 8 prognostic model genes and survival time. **(I–P)** Diagnostic ROC curves for the 8 prognostic model genes.

To identify independent prognostic factors, 8 genes (BAK1, ATG5, VEGFA, GABARAPL1, RSL1D1, CYCS, TOMM20, PGAM5) and clinical variables potentially associated with high risk were included in a multivariate Cox regression analysis. Variables with p < 0.1 in univariate Cox regression were selected for inclusion, resulting in the exclusion of clinical stage and gender. A prognostic nomogram was generated to visualize the independent prognostic factors, and its reliability was validated using calibration curves. The results indicated that clinical stage (I), age (>60 years), BAK1, GABARAPL1, and CYCS were potential prognostic risk factors. However, only age, BAK1, and GABARAPL1 remained independent prognostic factors, with GABARAPL1 demonstrating the most pronounced negative impact on OSCC patient outcomes (**Figures 4**, **5**). For each gene, we also constructed Kaplan-Meier survival curves and diagnostic ROC. The results indicated that high expression of the 8 autophagy and pyroptosis related genes was associated with shorter overall survival, further validating their prognostic significance. Additionally, 7 genes (excluding GABARAPL1, AUC = 0.523) exhibited strong diagnostic predictive capability, with BAK1 showing the highest diagnostic accuracy (AUC = 0.903) (**Figure 6**).

### 3.5 The LASSO-derived composite risk score exhibits favorable prognostic value

To reduce model complexity and improve interpretability, we calculated a combined risk score for each patient by applying a weighted linear combination of the expression levels of the eight prognostic genes, with weights derived from the LASSO-Cox regression coefficients. This risk score was then evaluated for its prognostic independence using univariate and multivariate Cox proportional hazards regression analyses. Clinical covariates including clinical tumor stage, age and gender were included in the multivariate model to control for potential confounding.

**Figure 7:**
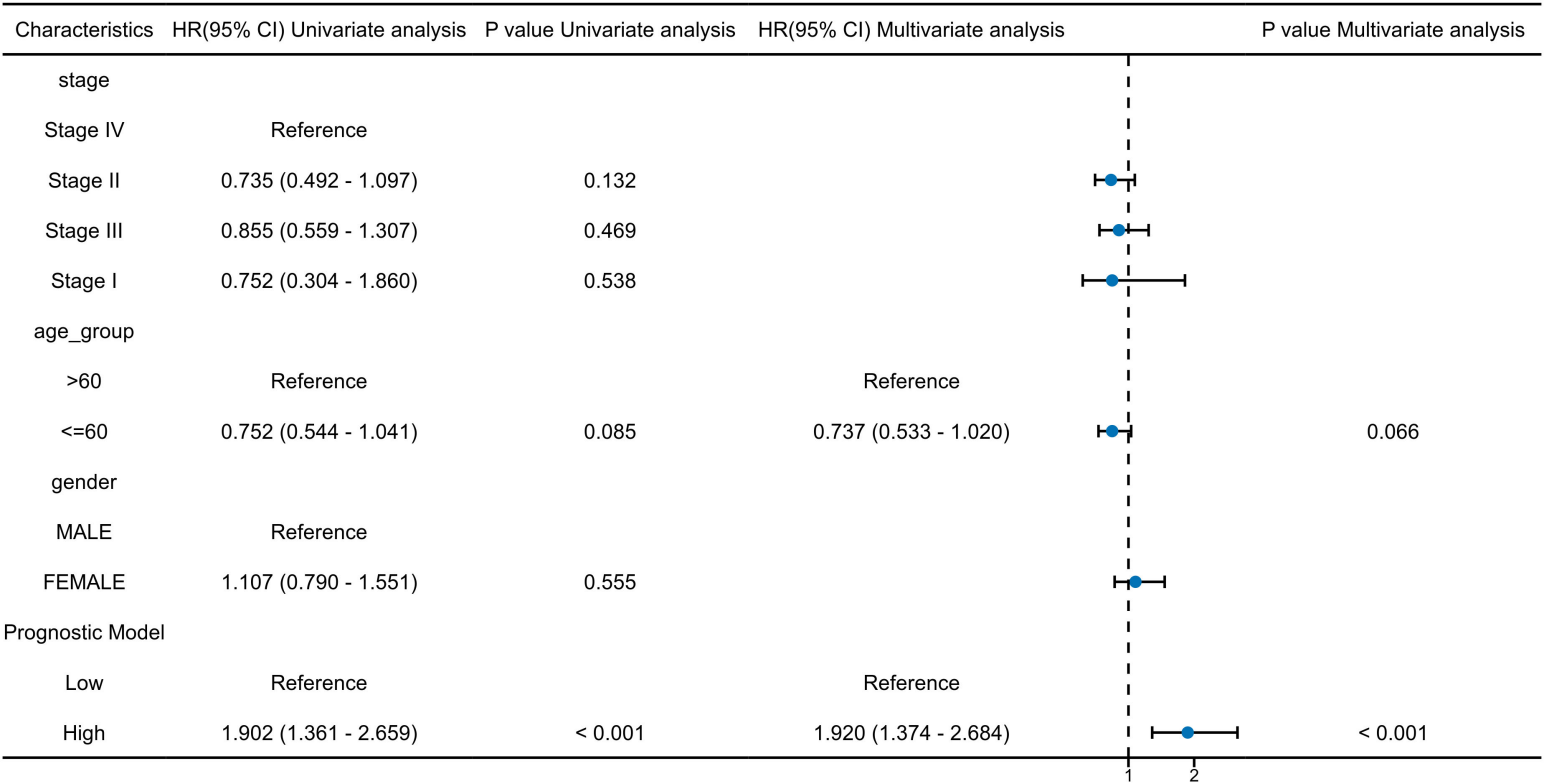
Univariate and multivariate cox regression analyses of the 8 prognostic model genes and key clinical factors.

In univariate Cox regression analysis, the combined risk score was significantly associated with overall survival in OSCC patients (HR = 1.902, 95% CI: 1.361–2.659, p < 0.001). Importantly, after adjusting for clinical covariates in the multivariate Cox model, the risk score remained a statistically significant independent prognostic factor (HR = 1.920, 95% CI: 1.374–2.684, p < 0.001). (**Figure 7**)These findings confirm that the 8-gene prognostic model retains great predictive power independent of clinical tumor stage, age or gender, thereby supporting its potential clinical utility in OSCC risk stratification.

### 3.6 External validation of model robustness

To assess the robustness and generalizability of the prognostic model, we performed external validation using an external cohort independent of the TCGA training data (GSE41613). Risk scores for each patient in the validation cohort were calculated using the LASSO regression coefficients derived from the original TCGA-based model. The median risk score was applied as a predefined cutoff to stratify patients into high- and low-risk groups.Kaplan-Meier survival analysis and Cox regression were conducted to evaluate the model’s discriminative ability in the external cohort. In addition, time-dependent ROC curves were generated to assess its prognostic accuracy over time.

**Figure 8:**
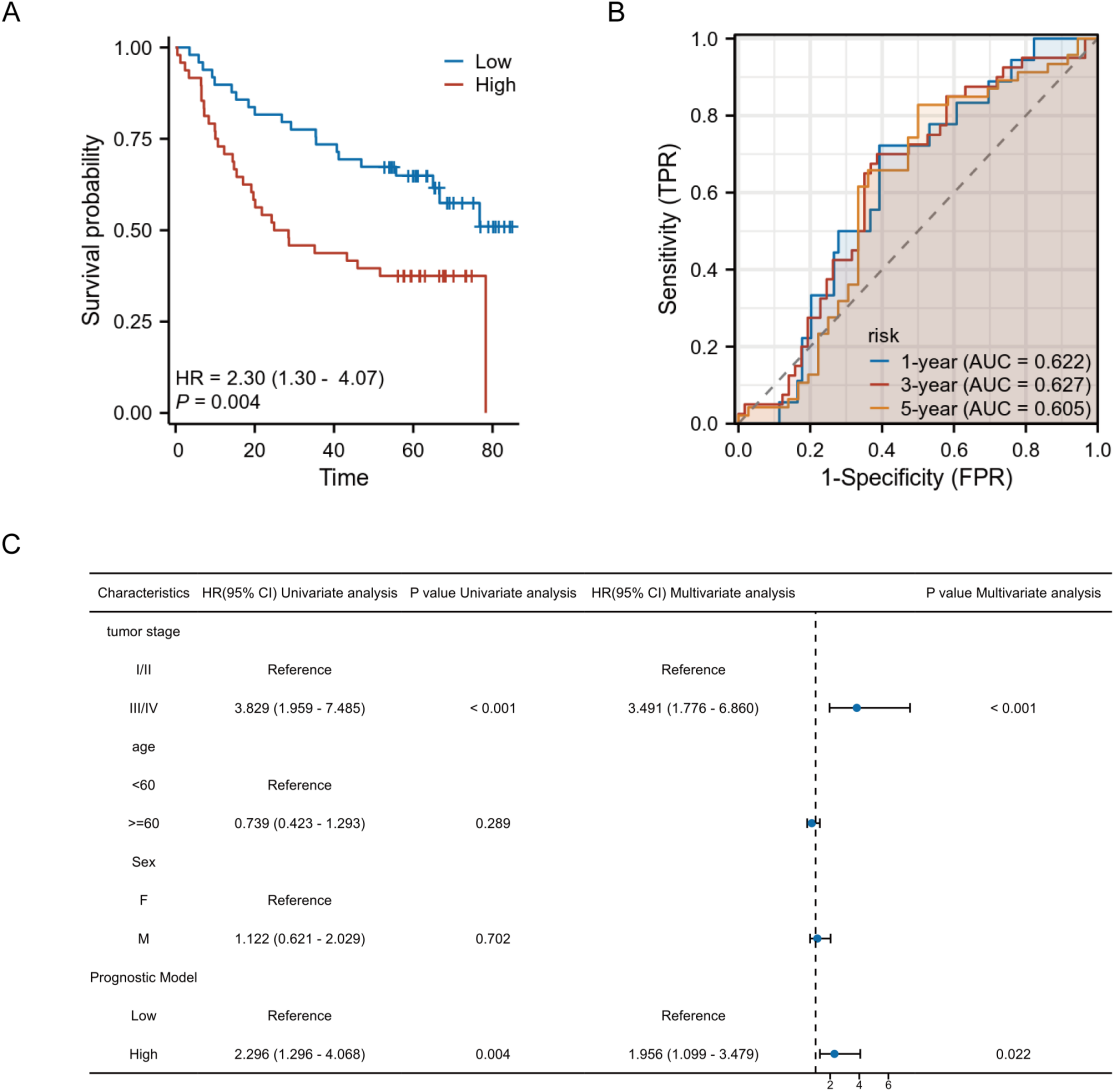
External validation of the prognostic model. **(A)** Kaplan-Meier survival curves show significantly worse survival in the high-risk group. **(B)** Time-dependent ROC curves demonstrate moderate predictive performance at 1-, 3-, and 5-year time points. **(C)** Forest plot of univariate and multivariate Cox regression analyses confirms the model’s independent prognostic value.

Kaplan-Meier survival analysis revealed a statistically significant difference in overall survival between the high-risk and low-risk groups, as stratified by the model. Patients in the high-risk group exhibited a markedly poorer prognosis compared to those in the low-risk group, with a hazard ratio (HR) of 2.30, supporting the model’s ability to effectively discriminate patient outcomes. (**Figure 8A**) Time-dependent ROC curve analysis further demonstrated the prognostic performance of the model across different time points. The area under the ROC curve (AUC) was 0.622 for 1-year survival, 0.627 for 3-year survival, and 0.605 for 5-year survival, indicating a consistent predictive capacity of the model over time. (**Figure 8B**) In the univariate and multivariate Cox regression analyses, the combined risk score derived from the prognostic model remained an independent predictor of survival after adjusting for other clinical parameters. Specifically, in multivariate analysis, high-risk patients had a significantly increased hazard of death, independent of tumor stage, age, and sex. Tumor stage also emerged as an independent prognostic factor, with advanced stages (III/IV) associated with significantly worse outcomes (**Figure 8C**).

Overall, these external validation results support the prognostic model as a statistically significant and independent predictor of survival in OSCC, reinforcing its translational potential in clinical risk stratification.

### 3.7 Expression and subcellular localization of autophagy and pyroptosis related genes

**Figure 9:**
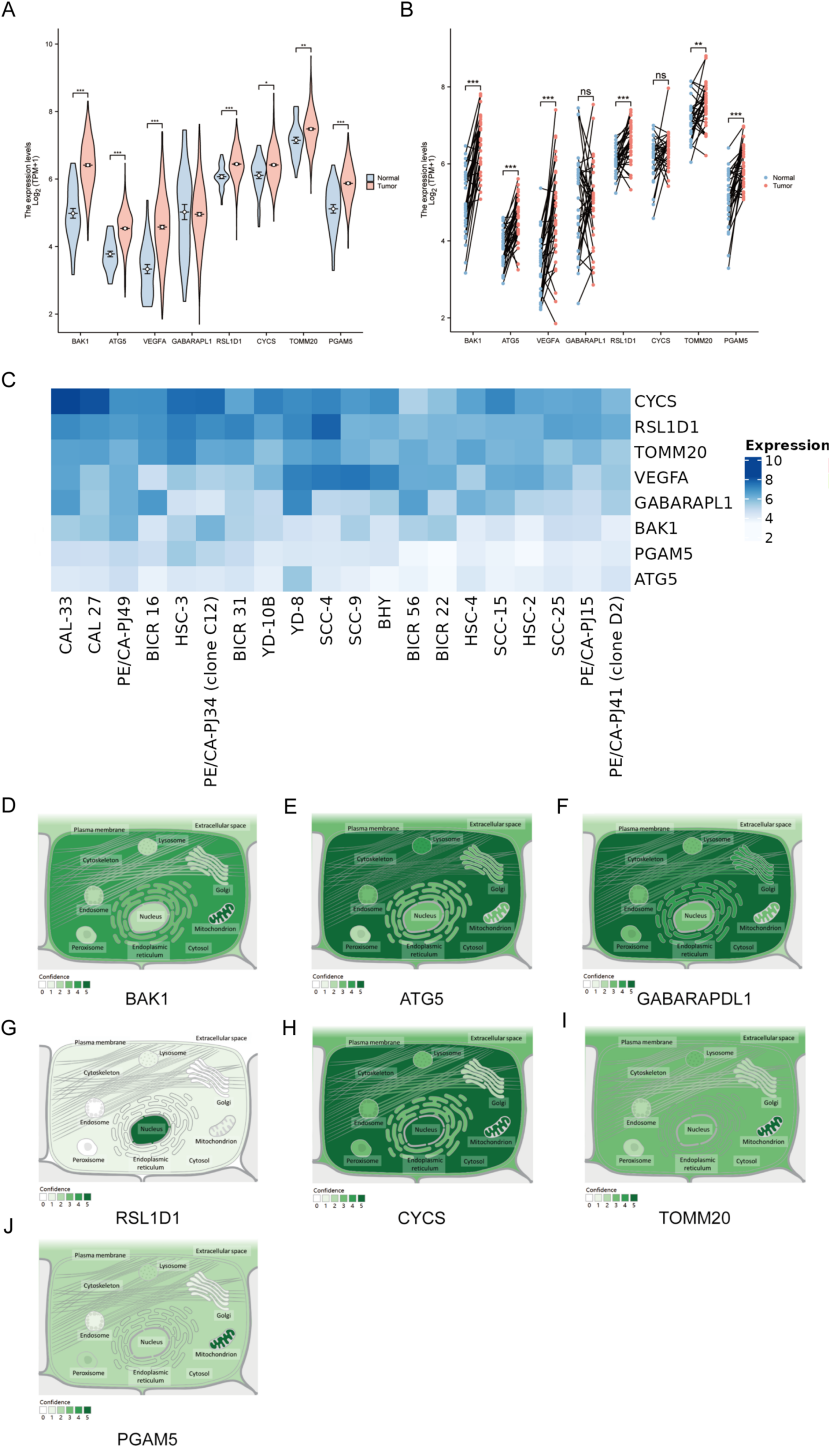
Expression and Subcellular Localization of Eight Prognostic Genes in the OSCC Model **(A)** Differential expression analyses of unpaired samples showing significant upregulation of 7 genes (BAK1, ATG5, VEGFA, RSL1D1, CYCS, TOMM20, PGAM5). **(B)** Paired sample analyses identified six genes (BAK1, ATG5, VEGFA, RSL1D1, TOMM20, PGAM5) showing marked upregulation in tumor tissues. **(C)**Expression heatmap of eight target genes in 20 OSCC cell lines, darker hues indicate higher fold-change expression. **(D–J)** Subcellular localization distributions of BAK1, ATG5, GABARAPL1, RSL1D1, CYCS, TOMM20, and PGAM5. Asterisks indicate statistical significance: *P < 0.05; **P < 0.01; ***P < 0.001. “ns” denotes not significant.

Compared to normal samples, the expression of all genes, except for GABARAPL1, was significantly higher in OSCC tumor tissues. Specifically, 7 genes (BAK1, ATG5, VEGFA, RSL1D1, CYCS, TOMM20, and PGAM5) exhibited elevated expression levels in OSCC tumors. Paired sample plots further revealed that GABARAPL1 and CYCS expression levels did not differ significantly among tumor, adjacent and normal tissues. In contrast, the remaining six genes (BAK1, ATG5, VEGFA, RSL1D1, TOMM20, PGAM5) displayed markedly elevated expression in OSCC tumors compared to paired adjacent or normal tissues (**Figures 9A, B**). Next, the heatmap more intuitively demonstrates universal upregulation of the eight genes across 20 OSCC cell lines (**Figure 9C**). The intracellular environment can be further partitioned into distinct organelles or regions, termed subcellular compartments. Mature proteins require specific subcellular organelles to exert stable biological functions, while subcellular structures provide relatively independent microenvironments for their functional execution. Subcellular localization aims to determine the precise intracellular location of target genes (e.g., nucleus, cytoplasm, endoplasmic reticulum, or plasma membrane), thereby elucidating their functional mechanisms. GeneCards provides comprehensive subcellular localization data; however, VEGFA localization information was unavailable in this study. Notably, except for RSL1D1, which was primarily localized to the nucleus, the remaining seven genes (BAK1, ATG5, VEGFA, CYCS, TOMM20, PGAM5, and GABARAPL1) were predominantly distributed in extranuclear cytoplasmic regions or concentrated within specific organelles. Notably, BAK1 is predominantly found in the mitochondria and cytoplasm, while ATG5 accumulates more in the cytoplasm, cytoskeleton, and lysosomal regions. GABARAPL1 was predominantly detected in the cytoplasm, Golgi apparatus, endoplasmic reticulum, and cytoskeleton. The CYCS gene was frequently localized to the nucleus but was also highly expressed in the cytoplasm, mitochondria, and lysosomes. TOMM20 is primarily localized in the mitochondria, with additional presence in the lysosome, cytoplasm, and endoplasmic reticulum. PGAM5 shares a similar distribution pattern with TOMM20, though it is detected at lower frequencies (**Figures 9D–J**). These localization patterns provide strong indications of the potential biological roles of these genes.

### 3.8 Gene set enrichment analysis

**Figure 10:**
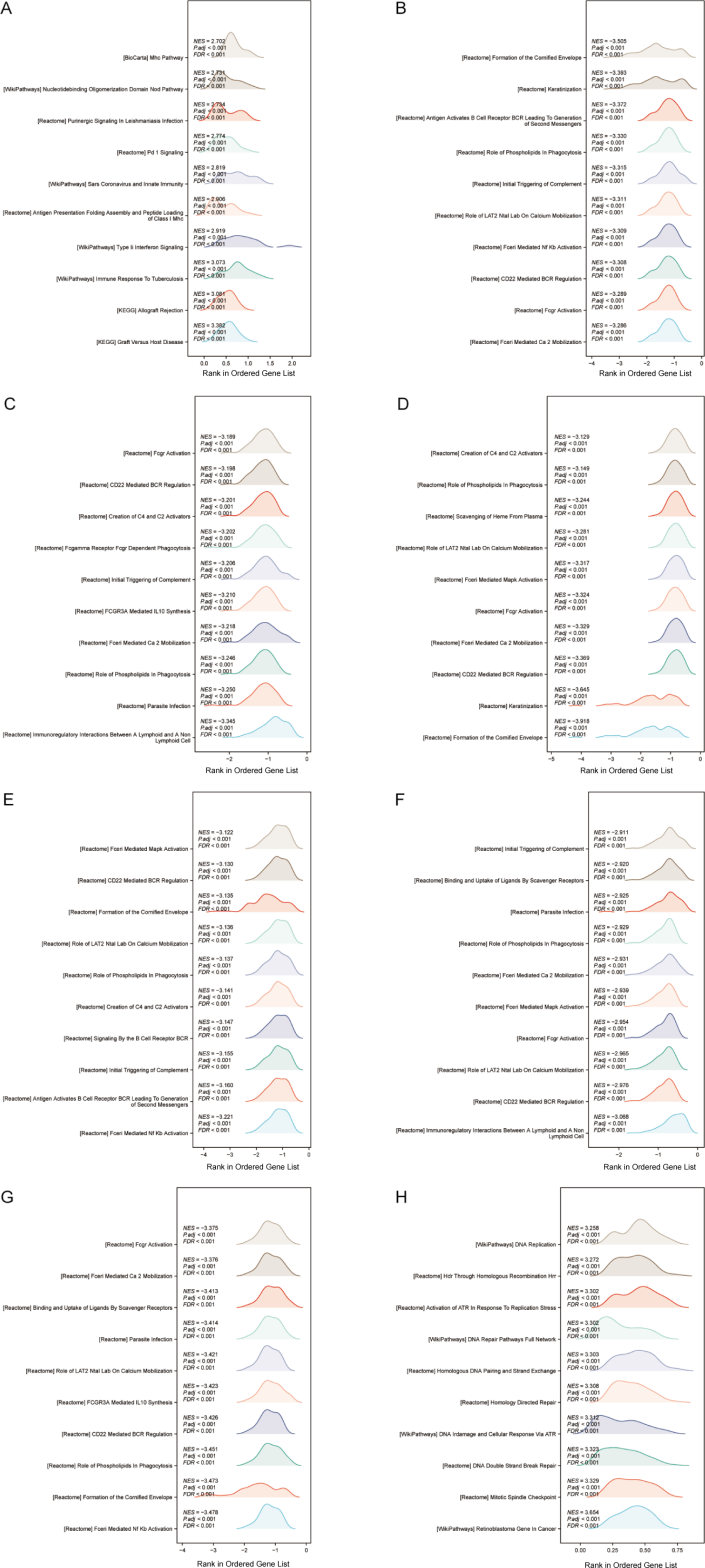
**(A–H)** GSEA of BAK1, ATG5, VEGFA, GABARAPL1, RSL1D1, CYCS, TOMM20, and PGAM5 was visualized as mountain plots.

To further study potential pathways through which autophagy and pyroptosis related genes influence OSCC prognosis, GSEA was performed individually for each of the 8 prognostic model genes. The top 10 pathways based on the absolute values of the enrichment scores were visualized. Among these, only BAK1 and PGAM5 exhibited positive enrichment scores, indicating their enrichment at the top of gene sets and upregulation in associated pathways. The remaining 6 genes were enriched at the bottom of gene sets. Notably, five pathways—Initial Triggering of Complement, Role of Phospholipids in Phagocytosis, CD22 Mediated BCR Regulation, Role of Lat2/NATL/LAB on Calcium Mobilization, and FceRI-Mediated Ca²^+^ Mobilization—were commonly enriched in at least five genes. Among these, complement activation may exhibit dual roles in the tumor microenvironment: excessive activation could induce chronic inflammation and promote OSCC progression, while its suppression might enhance immune evasion. Studies have demonstrated that elevated C5a levels correlate with tumor differentiation and OSCC expansion status, whereas C3a levels are associated with local tumor spread (57). Phospholipid metabolism plays a critical role in membrane remodeling, pathogen phagocytosis, and antigen presentation, which are essential for the functions of neutrophils and macrophages. Phospholipid metabolism-related genes, such as PLD1, have been identified to be key players in cancer stemness and chemotherapy resistance (58). CD22, an inhibitory co-receptor of the BCR, functions to maintain humoral immune homeostasis. Integrative analysis of RNA-seq datasets derived from The Cancer Genome Atlas (TCGA) repository revealed a significant positive association between elevated expression levels of B-cell and plasma cell-related genes and favorable survival outcomes across diverse malignancies, including head and neck cancer, melanoma, and ovarian cancer (59–61). LAB/NTAL/Lat2 is a transmembrane adapter protein closely related to LAT, capable of regulating signaling in almost all types of leukocytes (62). FCERI, a high-affinity IgE Fc receptor, mediates calcium influx that not only triggers mast cell degranulation but also activates calcineurin, a Ca2+ or calmodulin-dependent serine phosphatase calcineurin. Calcineurin dephosphorylates the nuclear factor of activated T cells (NFAT), which regulates T cell activation and is involved in thymocyte development, T cell differentiation, and self-tolerance (63). Given the recurrent identification of these pathways across multiple analytical approaches and their immunological relevance, we conducted immune infiltration analysis on OSCC samples (**Figure 10**).

### 3.9 Immune infiltration analysis of autophagy and pyroptosis related genes

**Figure 11:**
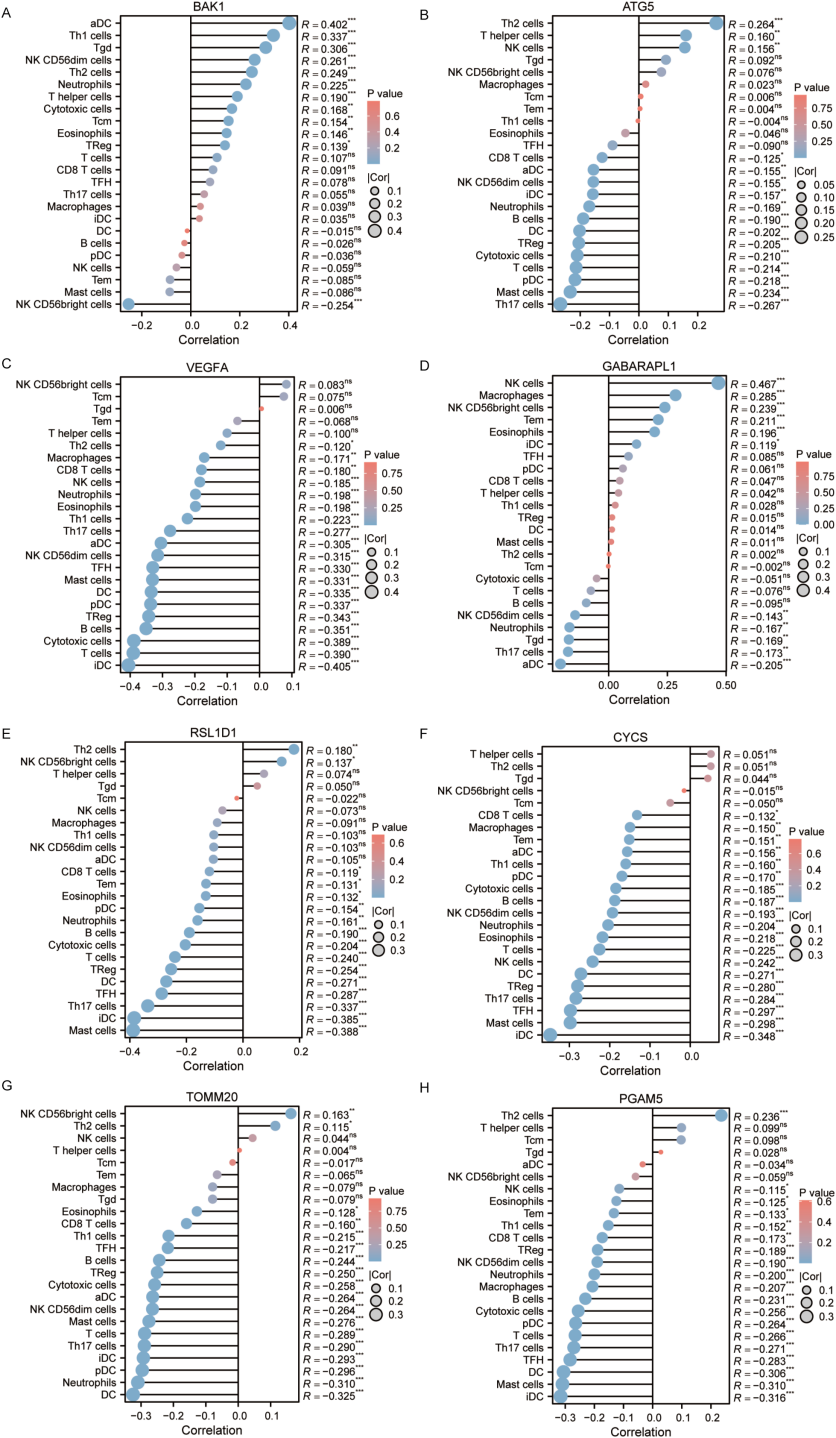
**(A–H)** Immune infiltration analysis of BAK1, ATG5, VEGFA, GABARAPL1, RSL1D1, CYCS, TOMM20, and PGAM5.

Autophagy, pyroptosis, inflammatory microenvironments, antitumor immunity, innate immunity, and malignant progression are closely associated with prognosis. Immune infiltration status, calculated based on markers of 24 immune cell types, was visualized via lollipop plots. Most of the 8 genes in the prognostic model were predominantly negatively correlated with immune infiltration. Interestingly, however, BAK1 and GABARAPL1—identified as independent prognostic factors—exhibited relatively positive correlations with immune cell infiltration (**Figure 11**).

### 3.10 Knockdown of BAK1 reduces proliferation and migration of OSCC cell lines

**Figure 12:**
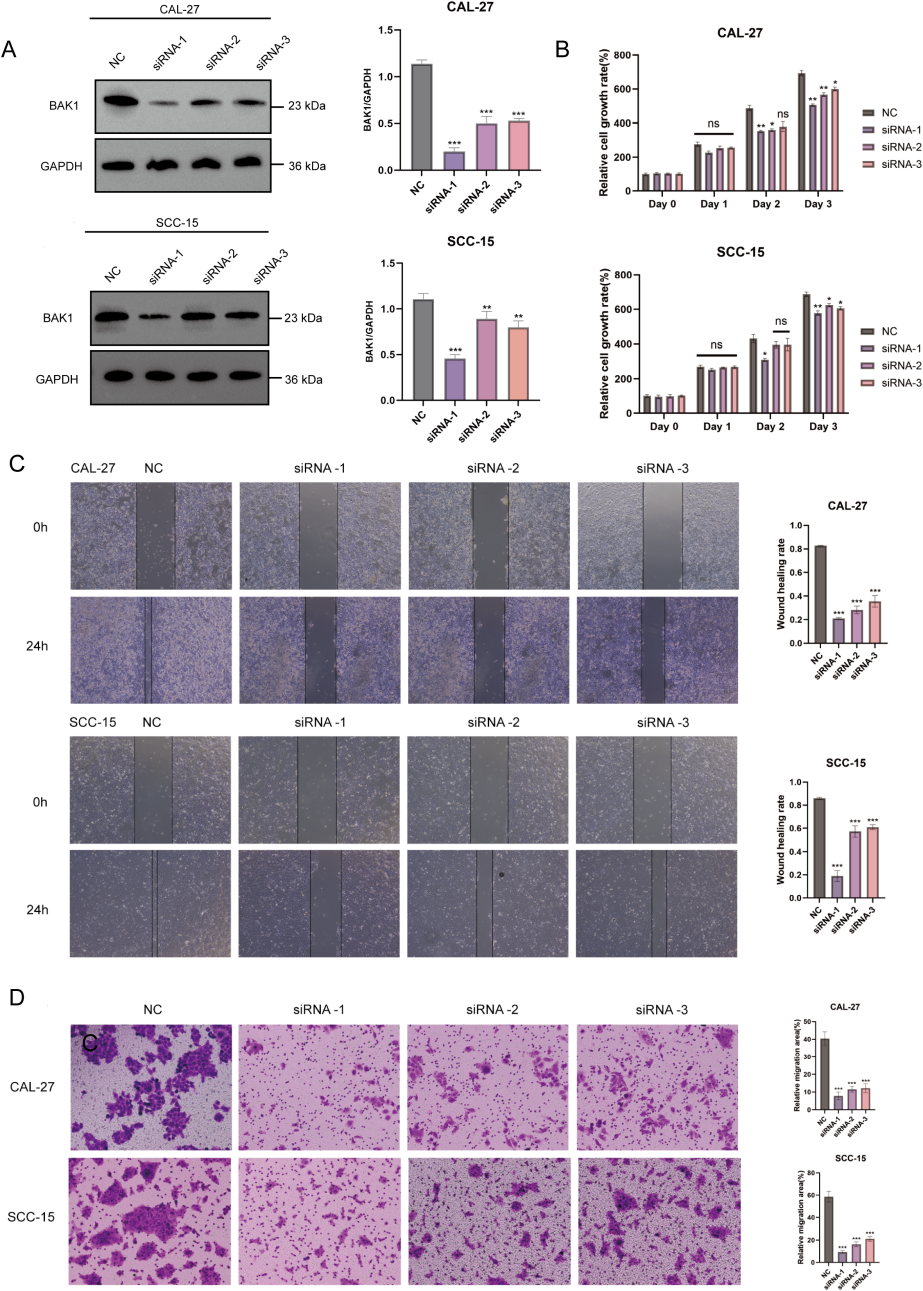
**(A)** Western Blot analysis demonstrated significant knockdown efficiency of BAK1 in CAL-27 and SCC-15 cell lines. **(B)** CCK-8 assays revealed reduced proliferation rates in BAK1-knockdown cells compared to the negative control (NC) group. **(C, D)** Wound healing and Transwell assays confirmed markedly impaired migration and invasion capabilities in BAK1-knockdown OSCC cells. The NC group (negative control) was transfected with non-specific siRNA using transfection reagents. All experiments were conducted in three independent replicates. Asterisks indicate statistical significance: *P < 0.05; **P < 0.01; ***P < 0.001. “ns” denotes not significant.

Due to its superior independent prognostic value and diagnostic predictive capability identified in prior analyses, BAK1 was selected as the representative gene for the prognostic model and subjected to *in vitro* experiments. siRNA was used to knock down BAK1 expression in CAL-27 and SCC-15 cell lines, and Western blot analysis was performed to assess knockdown efficiency. The results confirmed a clear reduction in BAK1 protein levels, indicating successful gene silencing. CCK-8 assays revealed that BAK1 knockdown significantly reduced cell proliferation compared to the negative control (NC) group. While no marked inhibition was observed on day 1, a gradual decline in proliferation became apparent on day 2, with statistically significant growth suppression in both cell lines by day 3. Furthermore, both wound healing assay and Transwell migration assay demonstrated that BAK1 knockdown significantly impaired the migratory capacity of OSCC cell lines, with the results achieving statistical significance (p < 0.001) (**Figure 12**).

## 4 Discussion

The global disease burden of OSCC remains substantial, with unfavorable 5-year survival rates (64). In recent years, research on autophagy and pyroptosis has uncovered increasing evidence of their close association with the initiation and progression of OSCC (65–68). Both autophagy and pyroptosis appear to exhibit dual roles in tumor biology—promoting or inhibiting cancer progression—depending on the microenvironmental context and triggering conditions (15, 49). Moreover, the complex regulatory relationships, pathways, and protein interactions between autophagy and pyroptosis, as well as their interplay with tumors, remain poorly understood (13). Previous bioinformatics studies have typically focused on establishing prognostic models using only autophagy-related genes (69, 70) or pyroptosis-related genes (50), often overlooking the potential information that could be derived from their complex interactions. Therefore, the present study aims to construct a prognostic model for OSCC using integrating autophagy and pyroptosis intersection genes through bioinformatics approaches. The goal is to guide prognosis prediction in OSCC patients and provide a foundation for potential individualized gene-targeted therapeutic strategies in clinical practice.

We identified 16 prognosis-related genes in OSCC by intersecting genes obtained from bulk survival regression analysis with autophagy-related and pyroptosis-related genes in this study. A prognostic model was then constructed using LASSO regression. Based on the TCGA dataset, the model’s stratification capacity and prognostic performance were evaluated through risk plots, survival analysis, and time-dependent ROC curves.

To enhance clinical interpretability, facilitate external validation, and avoid introducing excessive covariates into multivariate Cox regression, we calculated a combined risk score by applying a weighted linear combination of the expression levels of eight prognostic genes, with weights derived from the LASSO regression coefficients. After adjusting for conventional clinical variables such as tumor stage, age, and gender, the risk score remained a significant independent predictor of survival. External validation using the GSE41613 dataset further confirmed the robustness and generalizability of the model in an independent cohort. These findings support the potential clinical value of the risk model and provide a foundation for future studies integrating molecular features into standard prognostic assessment in OSCC.

The prognostic model derived in this study incorporated 8 genomic biomarkers (BAK1, ATG5, VEGFA, GABARAPL1, RSL1D1, CYCS, TOMM20, PGAM5), whose high expression was associated with unfavorable OSCC prognosis. High expression of BAK1 is associated with immune and inflammatory responses in OSCC patients, potentially mediated through the NOD-like receptor signaling pathway (71). Elevated ATG5 expression correlates with higher tumor grade, increased tumor size, advanced clinical stage, and lymph node metastasis in OSCC (47). VEGFA promotes OSCC cell proliferation and tumorigenesis (72, 73). RSL1D1 has been predominantly studied in colorectal cancer. However, its involvement in cellular senescence, proliferation, autophagy, and ferroptosis suggests a potential role in other tumor types, which warrants further investigation (74, 75). Upregulation of CYCS has been associated with larger tumor volumes and identified as a negative prognostic marker during cancer treatment (76). TOMM20 expression is minimal in normal oral tissues but is detected at varying levels in most tumor cells, regardless of differentiation status (77). PGAM5 has been implicated in promoting OSCC initiation, metastasis, and immune regulation, and may also participate in necroptosis (78). Among these, RSL1D1 was primarily localized to the nucleus, whereas the remaining seven genes were predominantly distributed in the cytoplasm, mitochondria, lysosomes, cytoskeleton, Golgi apparatus, and endoplasmic reticulum.

GSEA revealed that most enriched pathways were immune-related. The widespread detection of the Initial Triggering of Complement pathway in GSEA analysis suggests that the complement system may be closely involved in the development of OSCC. Complement participates through the classical, lectin, and alternative pathways, promoting tumor progression both via direct autocrine effects and indirect immune modulation (79, 80). Recent studies have revealed the prognostic value of complement components as biomarkers in squamous cell carcinoma, highlighting their complex, dual roles within the tumor microenvironment (81, 82). Although the precise mechanisms remain to be fully elucidated, clear evidence of systemic complement activation has been observed in OSCC patients. This is reflected by significantly elevated serum levels of the anaphylatoxins C3a and C5a. Importantly, increased C5a concentrations may serve as potential biomarkers for tumor invasiveness and differentiation in OSCC (57). Elevated C5a promotes tumor progression, partly through a marked reduction in CD4+ and CD8+ T cell infiltration, while lower levels appear to exert opposing effects (83). Targeting complement factors to modulate the tumor microenvironment thus represents a promising therapeutic avenue for OSCC patients (84).

In parallel, the Role of Phospholipids in Phagocytosis pathway was also identified in GSEA analyses of six out of the eight genes. Phosphatidylserine (PS), a well-studied phospholipid, is recognized as a key regulator of the tumor microenvironment. The translocation of PS from the inner to the outer leaflet of the plasma membrane serves as an important “eat-me” signal during phagocytosis. In tumor cells, persistent externalization of PS is widely regarded as a mechanism facilitating immune evasion (85, 86). Furthermore, dysregulated phospholipid metabolism, exemplified by PRDX1 overexpression, has been shown to enhance OSCC cell proliferation, migration, and immune escape by inhibiting pyroptosis and autophagy. This contributes to high postoperative recurrence rates (87). Therapeutic strategies targeting PS or its receptors (PSR) have progressed into clinical trials. Knockdown of PRDX1 has been demonstrated to suppress OSCC proliferation and revealed mechanisms involving reactive oxygen species (ROS)-dependent inhibition of pyroptosis and ROS-independent suppression of autophagy, highlighting its potential as a therapeutic target.

The expression of the 8 genes in OSCC samples also exhibited positive or negative correlations with immune infiltration. The tumor’s behavior—whether it enhances or suppresses antitumor immunity—depends on the subtypes, numbers, and distribution of immune cells in the tumor microenvironment, as well as the state of other cells, including tumor cells and tumor-associated fibroblasts, and the inflammatory factors they secrete. OSCC is a highly immunogenic tumor, and increased immune cell infiltration may serve as a positive prognostic marker (88). In fact, the tumor immune microenvironment (TIME) in OSCC involves complex regulatory interactions among 22 types of immune cells. The interplay between CD8+ T cells and CD4+ T cells is thought to suppress OSCC progression, whereas M0 macrophages exhibit the opposite effect. Regulatory T cells (Tregs) play a critical role in immune evasion, while dendritic cells (DCs) and natural killer (NK) cells contribute significantly to antitumor immunity (89, 90).

Furthermore, immune cell infiltration or dysfunction within the tumor microenvironment (TME) can in turn influence the expression of certain genes. For example, a loss of FIP200, a FAK family-interacting protein, has been observed in naïve T cells of cancer patients, leading to impaired antitumor immunity. This loss results in translational repression of the BAK1 gene (91). Overall, the OSCC TME is characterized by alterations in immune cells, immune checkpoints, and TME homeostasis, facilitating immune evasion and promoting immunosuppression (92, 93). In recent years, immunotherapy has been increasingly applied in OSCC treatment, offering new hope for patients (94).

Additionally, another key finding of this study is that among the identified genes, BAK1 and GABARAPL1 emerged as independent prognostic indicators. When compared to GABARAPL1 (AUC = 0.523), BAK1 (AUC = 0.903) demonstrated excellent diagnostic and predictive capacity.

Consequently, BAK1 silencing was experimentally induced in two OSCC cell lines—CAL-27 and SCC-15—under *in vitro* conditions, with subsequent results demonstrating a statistically significant attenuation of cell proliferation and migratory capacity. BAK1, also known as BCL2 Antagonist/Killer 1, is a member of the BCL2 family, often referred to as the “guardian of the mitochondrial gate.” The mitochondrial membrane potential is precisely regulated by members of the BCL-2 family, a key modulator of mitochondrial homeostasis. BAK1 primarily reduces mitochondrial membrane potential through oligomerization on the mitochondrial surface, thereby promoting apoptosis. Specifically, upon receipt of cell death signals, oligomerization forms pores in the outer mitochondrial membrane. During this process, released apoptotic factors activate effector caspases and drive their proteolytic maturation (95–97). As a programmed cell death pathway mediated by the GSDM family, the initiation of pyroptosis also relies on the inflammatory caspase activity of the caspase family. This mechanism may suggest a potential role for BAK1 in pyroptosis. Furthermore, prior studies have highlighted the pivotal roles of BAX and BAK1 in the three major forms of cell death: apoptosis, necrosis, and autophagy (98). However, the specific regulatory mechanisms of BAK1 in autophagy and pyroptosis pathways in OSCC remain to be further investigated.

Notably, beyond OSCC, analysis of RNA-seq data from 33 tumor projects in the TCGA revealed that BAK1 expression is significantly upregulated in malignant tumors including BLCA, UCEC, and LUSC. Additional research indicates that this protein interacts with the tumor suppressor gene TP53 under cellular stress (as provided by RefSeq). In multiple bioinformatics studies, BAK1 has served as part of prognostic models, demonstrating critical predictive roles in common malignancies such as esophageal squamous cell carcinoma, lung adenocarcinoma, hepatocellular carcinoma, and breast cancer (99–102).

In addition, BAK1 appears to be a downstream target of various non-coding RNAs, including circular RNAs (circRNAs) and microRNAs (miRNAs). These complex regulatory networks are closely associated with tumor initiation, progression, and therapeutic resistance across multiple cancer types. For instance, exosomal circ-0051443 has been shown to promote BAK1 expression by sponging miR-331-3p, thereby suppressing the malignant behavior of hepatocellular carcinoma cells (103). Similarly, miR-125b directly targets BAK1 and contributes to breast cancer progression (104), while BAK1 repression mediated by specific miRNAs has been demonstrated to promote the growth of prostate cancer cells (105). More intriguingly, the miR-503-3p–BAK axis has been reported to regulate radiation-induced cell death in OSCC, ultimately contributing to the development of radioresistance (106). Although the precise role of BAK1 in OSCC tumorigenesis remains to be fully elucidated, these findings suggest that targeting upstream miRNAs may offer a promising strategy. Future studies are warranted to explore specific miRNAs regulating BAK1 in OSCC, with the potential to leverage its high diagnostic relevance for therapeutic intervention.

Another independent prognostic gene identified in this study, GABARAPL1, is a member of the Atg8 protein family, which is associated with autophagosomes. It guides autophagosome formation, degradation, and lysosomal fusion. Additionally, GABARAPL1 plays multifunctional roles in phagocytosis, cell motility, intracellular transport, and oncogenic/tumor-suppressive regulation. Studies have demonstrated its critical role in the metastatic cascade, though notably, this process is uncorrelated with invasion (107–111). Interestingly, research has indicated that hypoxia induces GABARAPL1 expression, which promotes the secretion of exosomes and growth factors, ultimately facilitating tumor angiogenesis and tumor growth (112). However, experimental evidence demonstrates that hypoxia significantly increases pyroptosis, and GABARAPL1 knockout further exacerbates this process, indicating that GABARAPL1 suppresses pyroptosis while promoting tumorigenesis under hypoxic conditions (113). Additionally, high GABARAPL1 expression has been linked to poor prognosis in TNBC and HNSC, yet paradoxically, elevated expression correlates with longer overall survival in certain cancers, including hepatocellular carcinoma and lymph node-positive breast cancer (109, 111, 114, 115). These findings highlight that the role of GABARAPL1 in cancer remains unclear and warrants further investigation.

This study integrated bioinformatics analysis with *in vitro* experiments to establish a prognostic model and validate its value, but several limitations remain. Although the prognostic model was externally validated using an independent cohort to support its generalizability, certain limitations should be acknowledged. The relatively wide confidence interval of the hazard ratio (with a lower bound close to 1) and the modest 1-year AUC value (0.655) suggest that the model currently exhibits only moderate prognostic accuracy and robustness. Accordingly, while it is effective in stratifying OSCC patients by relative risk, its capacity for precise individualized risk estimation remains limited. Furthermore, external validation was performed solely on the GSE41613 dataset, which includes RNA expression profiles from 97 HPV-negative OSCC patients. Additional validation across larger and more heterogeneous cohorts will be essential to enhance the model’s reliability and expand its clinical utility. In addition, while *in vitro* experiments validated the effects of key genes on OSCC cell proliferation and migration, the precise mechanisms by which these genes regulate autophagy and pyroptosis in OSCC, as well as their influence on immune infiltration, remain unclear.

Future studies will employ conditional gene knockout mouse models to dynamically assess how autophagy- and pyroptosis-related genes influence the tumor microenvironment. Further mechanistic investigations are also planned to clarify their roles in OSCC pathogenesis and clinical outcomes. We believe these in-depth investigations will elucidate the translational potential of programmed cell death-related genes as therapeutic targets for OSCC.

In conclusion, this study established an autophagy and pyroptosis related gene prognostic model comprising 8 genes. This model addresses potential gaps in previous studies that independently investigated autophagy and pyroptosis, while providing a novel predictive method and reference biomarkers for OSCC patient prognosis. We anticipate that this work will offer help to the mechanisms of autophagy and pyroptosis in OSCC pathogenesis and progression, ultimately contributing to the development of personalized therapeutic strategies for clinical OSCC management.

## Data Availability

Publicly available datasets were analyzed in this study. This data can be found here: The Cancer Genome Atlas (TCGA) database (https://portal.gdc.cancer.gov) the GeneCards database (https://www.genecards.org), the MSigDBCollections gene set database (https://www.gsea-msigdb.org/gsea/msigdb/index.jsp), and the Genotype-Tissue Expression Project (GTEx) (https://www.gtexportal.org).
